# Neophobia in 10 ungulate species—a comparative approach

**DOI:** 10.1007/s00265-021-03041-0

**Published:** 2021-06-23

**Authors:** Alina Schaffer, Alvaro L. Caicoya, Montserrat Colell, Ruben Holland, Lorenzo von Fersen, Anja Widdig, Federica Amici

**Affiliations:** 1 Behavioral Ecology Research Group, Institute of Biology, University of Leipzig, Leipzig, Germany; 2 Department of Human Behavior, Ecology and Culture, Research Group ‘‘Primate Behavioural Ecology’’, Max Planck Institute for Evolutionary Anthropology, Leipzig, Germany; 3 Department of Clinical Psychology and Psychobiology, Faculty of Psychology, University of Barcelona, Barcelona, Spain; 4 Institute of Neurosciences, University of Barcelona, Barcelona, Spain; 5 Zoo Leipzig, Leipzig, Germany; 6 Nuremberg Zoo, Nuremberg, Germany

**Keywords:** Neophobia, Ungulates, Personality, Dietary breadth, Social integration, Social group size

## Abstract

**Abstract:**

Neophobia (the fearful reaction to novel stimuli or situations) has a crucial effect on individual fitness and can vary within and across species. However, the factors predicting this variation are still unclear. In this study, we assessed whether individual characteristics (rank, social integration, sex) and species socio-ecological characteristics (dietary breadth, group size, domestication) predicted variation in neophobia. For this purpose, we conducted behavioral observations and experimental tests on 78 captive individuals belonging to 10 different ungulate species—an ideal taxon to study inter-specific variation in neophobia given their variety in socio-ecological characteristics. Individuals were tested in their social groups by providing them with familiar food, half of which had been positioned close to a novel object. We monitored the individual latency to approach and eat food and the proportion of time spent in its proximity. Using a phylogenetic approach and social network analyses, we showed that across ungulate species neophobia was higher in socially more integrated individuals, as compared to less integrated ones. In contrast, rank and sex did not predict inter-individual differences in neophobia. Moreover, species differed in their levels of neophobia, with Barbary sheep being on average less neophobic than all the other study species. As group size in Barbary sheep was larger than in all the other study species, these results support the hypothesis that larger group size predicts lower levels of neophobia, and confirm ungulates as a highly promising taxon to study animal behavior and cognition with a comparative perspective.

**Significance statement:**

In several species, individuals may respond fearfully to novel stimuli, therefore reducing the risks they may face. However, it is yet unclear if certain individuals or species respond more fearfully to novelty. Here, we provided food to 78 individual ungulates with different characteristics (e.g., sex, rank, social integration, group size, domestication, dietary breadth) in different controlled conditions (e.g., when food was close to novel or to familiar objects). Across species, we found that socially integrated individuals responded more fearfully in all species. Moreover, being in larger groups decreased the probability of fearfully responding to novelty.

**Supplementary Information:**

The online version contains supplementary material available at 10.1007/s00265-021-03041-0.

## Introduction

Neophobia has been defined as the fearful reaction to novel stimuli or situations (Greenberg and Mettke-Hofmann 2001; Mettke-Hofmann 2017). In several taxa, neophobia is known to provide crucial fitness benefits (e.g., Wilson et al. 1994; Boissy 1995; Gosling and John 1999; Wolf et al. 2007). More neophobic individuals, for instance, are less likely to consume novel food which could be toxic and may have a lower chance of encountering predators and competitors, as they are less explorative (e.g., Robertson 1982; Greenberg and Mettke-Hofmann 2001; Crane et al. 2020). However, neophobia also comes with costs, as it may reduce competitive abilities by for instance decreasing exploration of novel food sources (Cole and Quinn 2012) and the probability of innovation (Greenberg 2003; Carere and van Oers 2004; Smith and Blumstein 2008; Cole and Quinn 2012; Ferrari et al. 2015). Furthermore, neophobia may increase stress-related mortality, as neophobic individuals are more easily stressed in novel situations (Carere and van Oers 2004) and might have higher energetic costs for vigilance (see Crane et al. 2020). Therefore, neophobia may have complex implications for individual fitness (Smith and Blumstein 2008; Ferrari et al. 2015), and different individuals and species may find different ways to balance the benefits and costs linked to neophobia (Greenberg 2003).

At the individual level, for instance, neophobia might be linked to individual dominance rank. In social species, more dominant individuals usually have better access to resources (e.g., space, food, mates) as compared to subordinates (Arave and Albright 1976; Ellis 1995; Clarke and Faulkes 1997). Therefore, dominant individuals may gain lower potential payoffs from novelty and might be more neophobic than subordinates (Hegner 1985; Greenberg-Cohen et al. 1994; Lahti 1998; Laland and Reader 1999; Greenberg and Mettke-Hofmann 2001; Wolf et al. 2007). However, while some studies have found evidence that subordinates are less neophobic than dominant conspecifics (Katzir 1982; Di Bitetti and Janson 2001; Stahl et al. 2001; Boogert et al. 2006), at least in some contexts (e.g., Dingemanse and de Goede 2004), other studies have not (e.g., Boogert et al. 2006; Mettler and Shivik 2007; Moretti et al. 2015).

Similarly, social integration in the group might be linked to differences in neophobia. Strong social bonds, for instance, enhance individual fitness (e.g., Silk et al. 2003, 2009, 2010; Cameron et al. 2009; Schülke et al. 2010; Archie et al. 2014), and integration in the social group increases others’ tolerance over food (Amici et al. 2020; Dell'Anna et al. 2020). Therefore, individuals that are better integrated in the social network might also gain lower potential payoffs from novelty (as compared to less integrated group members) and thus be more risk-aversive and less prone to explore novelty (see Wolf et al. 2007).

Furthermore, neophobia might also differ between sexes. On the one hand, males show higher variance in reproductive payoff than females so that males should thus be less risk aversive and less neophobic than females (Cornwell-Jones and Kovanic 1981; Clutton-Brock 1988; Laviola and Loggi 1992; Schuett et al. 2010; Crane et al. 2020). On the other hand, males are often dominant over females, at least in mammals, and the positive link between rank and neophobia may be confounded by more specific sex effects (see Wingfield et al. 1987; Crane et al. 2020).

At the species level, less neophobic species may also be characterized by more generalist diets (Glickman and Sroges 1966; Clarke and Lindburg 1993), extractive foraging (Day et al. 2003), higher environmental variability (Greenberg 1984, 1990; Mettke-Hofmann et al. 2002; Martin and Fitzgerald 2005; Bergman and Kitchen 2009), and lower predation pressure (Crane and Ferrari 2017). More generalist species, for instance, feed on a higher number of food items with highly variable quality so that being less neophobic would allow them to more easily switch across resources and explore novel ones (Greenberg 1983; Greenberg and Mettke-Hofmann 2001; Visalberghi et al. 2002; Day et al. 2003). Furthermore, domesticated species might be less neophobic. By having been selected for their ability to live in close association with humans, domesticated animals might show less fearful responses to novel stimuli, as also suggested by recent studies in dogs and rats (Sheppard and Mills 2002; Kaulfuß and Mills 2008; also see Modlinska et al. 2015; Moretti et al. 2015).

Finally, group size may also explain differences in levels of neophobia. In species with larger group size, for instance, individuals are usually less vulnerable to predation and more easily benefit from social facilitation when interacting with novel food so that they might be overall less neophobic (Pulliam and Caraco 1984; Beck and Galef 1989; Galef et al. 1990; Visalberghi and Addessi 2000; Addessi and Visalberghi 2001; but see Stöwe et al. 2006; Addessi et al. 2007). However, group size might not necessarily have an impact in evolutionary terms. Several studies, for instance, suggest a direct effect of group size on neophobia in developmental terms, through direct experience (Brown et al. 2013; Modlinska and Stryjek 2016). Ravens (*Corvus corax*), for example, show different reactions to novel objects depending on whether they are tested alone or in groups (Stöwe et al. 2006). Some studies have indeed found a link between living/being tested in larger groups and showing reduced neophobia within different species (Heinrich and Marzluff 1991; Visalberghi and Addessi 2000; Lonsdorf 2006; Tarnaud and Yamagiwa 2008; Costa et al. 2014; Moretti et al. 2015). However, others have found little to no evidence (Ryer and Olla 1991; Brown and Laland 2001, 2002; Stöwe et al. 2006; Apfelbeck and Raess 2008; Dardenne et al. 2013).

In this study, we aimed to study inter-individual and inter-specific variation in neophobia in ungulates. Ungulates are a largely neglected taxon in comparative psychology, despite their high variation in socio-ecological characteristics, which makes them an ideal candidate to test how specific socio-ecological conditions may favor the emergence of certain traits or behaviors (Caicoya et al. in review; see Shultz and Dunbar 2006; Schaffer et al. 2020). In this study, we tested neophobia toward novel objects (see Greenberg 1992; Mettke-Hofmann et al. 2002; Greenberg 2003; Brown and Jones 2016) by providing ten ungulate species with familiar food, half of which had been positioned close to a novel object. Our study species differed in their socio-ecological characteristics and in particular in terms of dietary breadth, group size and domestication (see “Methods” section; see Table 1). All species were tested in captivity: While captive individuals are usually expected to be less neophobic than their wild counterparts (Bergman and Kitchen 2009; van de Waal and Bshary 2010; Benson-Amram et al. 2013; but see Crane and Ferrari 2017), neophobia is also known to have a strong genetic component (Mettke-Hofmann 2017). Hence, testing captive individuals should reproduce “consistent and meaningful differences among species according to their evolutionary history” (see Crane et al. 2020, p.220). Based on existing literature, we expected differences in neophobia both within and across species. In particular, we predicted that neophobia should be higher in more dominant individuals (Prediction 1), in individuals that are better integrated in their social group (Prediction 2) and in females (Prediction 3). Moreover, we predicted that neophobia should vary across species, being higher in species with lower dietary breadth (Prediction 4), living in smaller groups (Prediction 5), and/or having been domesticated (Prediction 6).

**Table 1:** Socio-ecological characteristics of the species tested (in bold, those showing a significance preference for the novel side)

Species	Dietary breadth (wild)	Group size (wild)	Actual group size	Domestication
**Barbary sheep**	69–79^1^	5–25^2^	15	No
Dromedary	17–58^3^	2–20^4^	7	Yes
Giraffe	93^5^	1–46^6^	6	No
Goat	33–126^7^	5–100^8^	7–9	Yes
Guanaco	35–76^9^	2–20^10^	4	No
Lama	> 35^11^	16^12^	4	Yes
Oryx	45^13^	10–30^12,14^	5	No
Przewalski horse	52^15^	< 10^16^	4	No
Red deer	145^17^	4–10^18^	7	No
Sheep	29–79^7,8,19^	2–60^20,21^	10	Yes

^1^Ogren 1962, Ramsey and Anderegg 1972; ^2^Gray and Simpson 1982; ^3^Elmi et al. 1992, Am Abbas et al. 1995; ^4^Gauthier-Pilters and Dagg 1981; ^5^Berry and Bercovitch 2017; ^6^Muller et al. 2018; ^7^González-Pech et al. 2015, Mellado 2016; ^8^Nowak and Paradiso 1983; ^9^Puig et al. 2001, Baldi et al. 2004; ^10^Bank et al. 2002, Marino and Baldi 2008; ^11^Posse and Livraghi 1997; ^12^Nowak and Walker 1999; ^13^Gilbert and Woodfine 2004; ^14^Newby 1984; ^15^Slivinska and Kopij 2011; ^16^Grum-Grzhimailo 1982; ^17^Gebert and Verheyden‐Tixier 2001; ^18^Gibson and Guinness 1980, Clutton-Brock et al. 1982; ^19^Fox and Streveler 1986; ^20^McClelland 1991; ^21^Maisels 1993

## Methods

### Subjects

We studied 78 subjects belonging to 10 ungulate species across three years. Subjects were housed in their natural groups at the zoos of Barcelona (Spain), Barben (France), and Nuremberg and Leipzig (Germany) and were all individually recognizable. We tested one group of 5 oryx (*Oryx dammah*) in Barcelona; one group of 7 dromedaries (*Camelus dromedarius*) and one group of 7 red deer (*Cervus elaphus*) in Barben; one group of 15 Barbary sheep (*Ammotragus lervia*) in Nuremberg; one group of 6 giraffes (*Giraffa camelopardalis rothschildi*), 2 groups of goats (*Capra aegagrus hircus*), one with 9 and one with 7 individuals, one group of 4 guanacos (*Lama guanicoe*), one group of 4 lamas (*Lama glama*), one group of 4 Przewalski horses (*Equus ferus przewalskii*) and one group of 10 sheep (*Ovis aries*) in Leipzig. For the analyses, we had to remove four subjects (i.e., two goats and two sheep) for which we had no behavioral information (as the individuals were removed from their groups during the study and observations could not be completed). Therefore, the final study sample was N = 74. None of the study subjects had ever been tested in a neophobia test before, and none had, to the best of our knowledge, come in contact with objects with the same shape and color as the ones used in this study, although all species occasionally participated in enrichment activities. None of the study subjects had ever participated in an experimental task, except for 3 of the 6 giraffes, which had participated in (i) a task on physical cognition in which they had been exposed to two small plastic containers (~ 15 × 15 × 3 cm) that could contain food (Caicoya et al. 2019), (ii) a quantity discrimination task in which they had been tested with two white trays containing food (Caicoya et al. 2020), and (iii) an inhibition task in which they had been exposed to a plastic cylinder with food (ALC et al., unpublished data).

All groups included males and females of different age and ranks (see Online Resource, Table S1) and differed in their socio-ecological characteristics, including dietary breadth, social group size, and domestication (see Table 1). To classify our study species according to their dietary breadth, social group size, and domestication, we used data from literature (see references in Table 1). However, these studies were conducted with different procedures and in very different conditions so that we considered inappropriate to calculate species-specific indexes and use them as direct test predictors in the models (see below), as they were not strictly comparable. Dietary breadth, for instance, may be measured in terms of how many plant species are eaten (i.e., taxonomic dietary diversity) or how many plant lineages (i.e., phylogenetic dietary diversity), but these measures are not positively correlated (Kartzinel and Pringle 2020). Moreover, even if the same index is used, methodological differences in the way data are collected (e.g., observational effort, sampling areas) can importantly affect the results of these categorizations. In the models, we therefore tested for inter-specific differences (including species as test predictor) and then interpreted the results based on the socio-ecological information on the species, as available from literature. Furthermore, as group size might affect neophobia independently of evolutionary history (see above), we also included the actual size of our study groups as a possible explanation of differences in neophobia (see Table 1).

### Behavioral observations

We conducted behavioral observations on each study group to determine the dominance rank and the social integration of each individual. Throughout the study period, we recorded via all occurrence sampling all dyadic agonistic interactions with a clear winner-loser outcome (i.e., threat, chase, fight) for each species (Altmann 1974). We assessed dominance hierarchy using the Elo method (Neumann et al. 2011) and, in particular, the EloRating package, version 0.43 in R (version 3.5.0, R Core Team 2018). We set 1000 as the individual start values and 100 as the k factor, which is a weighted constant based on winning probability (Albers and de Vries 2001; Sánchez-Tójar et al. 2018). We then averaged these values through the study periods and standardized them to range from 0 (i.e., lowest rank) to 1 (i.e., highest rank). Below, we refer to these values simply as Elo-ranks (Table S1). For more studies using the Elo method, see for instance Gomez-Melara et al. (2021) or Langos et al. (2013). For the giraffes and two of the red deer, we observed no agonistic interactions throughout the study period. For these individuals, rank was assessed by the experimenter together with the animal keepers, based on observations of priority of access to food (i.e., ranking all the giraffes from 1 to 6, and the two red deer from 1 to 7, and then rescaling the ranks to be between 0 and 1).

In each group, we further assessed Eigenvector centrality as a measure of individual social integration. For this reason, we determined the spatial proximity network in each study group, based on observational data collected with 100 instantaneous scans per group. Scans were made every 15 min across several days and recorded the spatially closest individual (“nearest neighbor”) of each group member (Altmann 1974). We built an undirected weighted matrix for social network analyses, which were run using the following packages in R: vegan (version 2.5–3; Oksanen et al. 2018), asnipe (version 1.1.10; Farine 2013), and igraph (version 1.2.1; Csardi and Nepusz 2006). Social network analyses assessed individuals’ Eigenvector centrality (Table S1), which is a measure proportional to the sum of the centralities of each individual’s neighbors and measures the importance of individuals as “social hubs” (Farine and Whitehead 2015; Farine 2017). As multiple researchers conducted behavioral observations, we ensured inter-observer reliability by starting data collection only after reaching inter-observer reliability > 90%, as estimated by comparing multiple random samples of behavior (Kaufman and Rosenthal 2009).

### Neophobia test

In all species, we administered the neophobia task in a familiar environment, testing all subjects together in their study group, in their outer enclosure. In the neophobia task, we included two different phases, the habituation phase (consisting of two sessions) and the experimental phase (consisting of two further sessions). All sessions were administered in different days, to reduce the effect of other contingencies on individual response. In the habituation phase, we placed preferred familiar food in two familiar locations, approximately 2 m from each other (although this distance was slightly increased/reduced depending on the animal size). The position of the two food locations was the same through all trials in each species, but we waited to place all items (and therefore to start the trial) until all animals were further than 1 m from both locations. As animals in all study groups had visual access to the set-up, sessions started when the food (and the novel object) had been positioned. To ensure high motivation, we used familiar food that was highly preferred by the study subjects. In the experimental phase, we repeated exactly the same set-up, but close to one of the two food locations (i.e., approximately 1 m, although this distance was increased/decreased depending on the animal size), we also positioned one visible novel object (i.e., either a plastic red bucket or a plastic blue bowl, either right or left, depending on the session, approximately 20 × 20 × 40 cm and 30 × 30 × 20 cm, respectively). We administered two sessions for each phase and study group, starting with the same object for all species to increase comparability. Each session lasted 10 min or until the food in one of the two locations was consumed. We used two different novel objects instead of two repeats of the same object, to create more accurate measures of novelty response (see Greggor et al. 2015), and we applied short sessions to avoid habituation to the novel object (Greenberg and Mettke-Hofmann 2001). For Barbary sheep, we used other objects instead (i.e., a plastic red ball and a plastic blue bucket, with the same dimensions as the objects above), as the keepers already used objects similar to the ones used for the other species during their daily feeding routine. For oryx, we administered only one experimental session (as the coronavirus outbreak did not allow us to complete testing). Although we originally aimed to use novel food to measure individual levels of neophobia, we had to use familiar food and novel objects in order to comply with the procedural recommendations of the zoos in which data were collected.

### Coding

We video-recorded all sessions. From the videos, we coded the identity of each individual approaching the food (i.e., individual latency to approach with the muzzle within 1 m from the food), the time spent in proximity of the food (i.e., from the time approaching the food to the time moving more than 1 m away from the food), and the latency to eat the food (i.e., from the moment the subject first approached it). In the experimental phase, we also further specified the food approached (i.e., familiar or novel). We then prepared our datasets, entering six lines per individual, one for each of the two sessions of the habituation phase, and two for each of the two sessions of the experimental phase (for each session, one line for the familiar food and one for the food close to the novel object). For each line, we entered the individual latency to approach food for the first time in the session, the individual latency to eat the food for the first time in the session, the total time the individual spent in proximity of the food in the session, and the time the individual did not spend in proximity. We further specified the subject identity, its species, sex, rank and centrality (i.e., social integration, see above), the session number, trial duration, and whether the food approached was familiar or novel. If subjects never approached the food in one session, we assigned them the total duration of the trial as latency (i.e., 600 s), as often done in literature on neophobia (e.g., Greggor et al. 2016). By simultaneously presenting food close to a novel object or not, we could avoid order effects and reduce the possibility that our measure was an artefact of motivation (as both kinds of food were available close to each other and at the same time). To calculate inter-observer reliability, the last author recoded 20% of the recorded videos (i.e., 9 of the 44 sessions recorded in the 11 study groups). Inter-observer reliability was excellent (i.e. Spearman exact correlation for latency to approach food, N = 97, rho = 0.999, *p* < 0.001; for latency to eat food, N = 97, rho = 0.984, *p* < 0.001; for time spent in proximity, N = 97, rho = 0.995, *p* < 0.001). It was unfortunately not possible to analyze data blind, because our study included (i) behavioral observations of focal animals during their daily interactions, which were coded live, and (ii) a neophobia test that was subsequently coded from the videos, in which the presence and side of the novel object was clearly visible.

### Statistical analyses

Analyses were conducted using generalized linear mixed models (Baayen et al. 2008) with the MCMCglmm package (version 1.0.1; Hadfield and Nakagawa 2010) in R (version 3.5.0, R Core Team 2018). To control for phylogenetic relationships across study species, we used the package ape (Paradis and Schliep 2019) to build a consensus tree from 10,000 trees, which had been subsampled and pruned from the mammal tree of life to match our study species (Upham et al. 2019). In all models, we then included a covariance matrix with the phylogenetic relationship between species, as based on the consensus tree (for a similar approach, see e.g. Lukas and Clutton-Brock 2017, 2020; Lukas and Huchard 2019). All models were run with a Gaussian distribution and non-informative priors, using 1,000,000 iterations, a burn-in of 100,000, and a thinning interval of 300 to facilitate convergence and minimize autocorrelation (see e.g. Lukas and Clutton-Brock 2017; McElreath 2020). We repeated all the analyses three times, visually inspected the models for convergence, and found no evidence of convergence issues. We considered terms to be statistically significant when the pMCMC values were lower than 0.05 (see e.g. Lukas and Clutton-Brock 2017).

We conducted three different models, assessing whether latency to approach food (Model 1), latency to eat food (Model 2), and time spent in proximity of food (Model 3) varied across species and individuals, depending on the side approached (i.e., close/opposite to the novel object; hereafter, novelty). In particular, we assessed whether latency to approach food (Model 1), latency to eat food (Model 2), and time spent in proximity of food (Model 3) were predicted by the 2-way interactions of novelty with individual rank (Prediction 1), novelty with individual centrality (Prediction 2), novelty with sex of subject (Prediction 3), and novelty with species (Predictions 4–6). Two-way interactions also included interaction terms as main effects. In all models, we further controlled for session number and duration (in Model 3, as offset term) and included subject identity as random factor. In case of significant categorical predictors with more than two categories (i.e., when the interaction between novelty and species was significant), we conducted post-hoc tests with the emmeans package (version 1.5.0, Lenth et al. 2020).

## Results

In Model 1, after accounting for phylogeny, we only found a reliable effect of the 2-way interaction of novelty with centrality on the latency to approach food (posterior estimate: 267.4 [95% confidence intervals, CIs: -1.6 to 511.8], *p* = 0.046). In particular, more central individuals had a higher latency to approach the novel side (as compared to the familiar one), while the pattern reversed for less central individuals, which had a much higher latency to approach the familiar side. Rank and sex had no effect on the latency to approach food (neither in interaction with novelty nor as main effects; rank, posterior estimate -51.9 [95% CIs: -136.7 to 49.0], *p* = 0.243; male sex, posterior estimate 66.9 [95% CIs: -3.7 to 134.3], *p* = 0.059). Moreover, none of the species differed in the latency to approach the familiar versus the novel side (post hoc tests: Barbary sheep, posterior estimate 101.4 [highest posterior-density intervals, HPDs: -2.3 to 208.5]; dromedary, posterior estimate 83.4 [HPDs: -37.9 to 191.8]; goat, posterior estimate 60.2 [HPDs: -62.6 to 180.8]; red deer, posterior estimate -72.8 [HPDs: -204.8 to 48.8]; Przewalski horse, posterior estimate 13.6 [HPDs: -175.6 to 188.9]; giraffe, posterior estimate 84.4 [HPDs: -68.8 to 233.4]; lama, posterior estimate 1.1 [HPDs: -167.2 to 171.4]; guanaco, posterior estimate 169.4 [HPDs: -5.6 to 344.7]; oryx, posterior estimate 54.4 [HPDs: -138.2 to 253.3]; sheep, posterior estimate 48.3 [HPDs: -95.1 to 187.4]). Session number had no significant effect on the latency to approach food (posterior estimate: -15.9 [95% CIs: -45.7 to 19.0], *p* = 0.357).

After accounting for phylogeny in Model 2, we found no significant effect of rank, centrality, or sex on the latency to eat food (neither in interaction with novelty nor as main effects; rank, posterior estimate -41.0 [95% CIs: -135.5 to 56.0], *p* = 0.395; centrality, posterior estimate -146.5 [95% CIs: -374.5 to 88.4], *p* = 0.227; male sex, posterior estimate 69.9 [95% CIs: -4.7 to 151.2], *p* = 0.080). Moreover, none of the species differed in the latency to eat food on the familiar versus the novel side (post hoc tests: Barbary sheep, posterior estimate 80.4 [HPDs: -29.3 to 190.0]; dromedary, posterior estimate -61.5 [HPDs: -171.9 to 55.2]; goat, posterior estimate -33.2 [HPDs: -155.5 to 94.5]; red deer, posterior estimate -84.6 [HPDs: -217.8 to 56.5]; Przewalski horse, posterior estimate 71.3 [HPDs: -111.1 to 257.2]; giraffe, posterior estimate 117.3 [HPDs: -58.0 to 274.5]; lama, posterior estimate 127.3 [HPDs: -56.6 to 305.4]; guanaco, posterior estimate 30.7 [HPDs: -141.3 to 196.5]; oryx, posterior estimate 50.4 [HPDs: -150.7 to 249.7]; sheep, posterior estimate -6.5 [HPDs: -143.0 to 139.2]). Session number had no significant effect on the latency to eat food (posterior estimate: 27.4 [95% CIs: -5.1 to 62.1], *p* = 0.113).

**Fig. 1:**
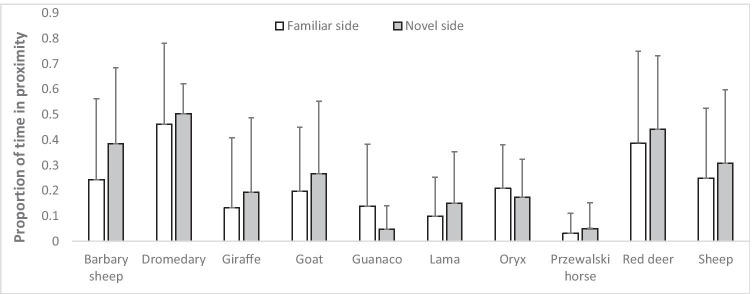
For each species, mean proportion of time spent (+ SE) close to food on the familiar side (i.e., with no object: white bars) and on the novel side (i.e., with the novel object: grey bars)

Finally, after accounting for phylogeny in Model 3, we found a significant effect of rank (posterior estimate: 84.8 [95% CIs: 19.7 to 146.6], *p* = 0.013) and centrality (posterior estimate: 201.4 [95% CIs: 41.0 to 354.2], *p* = 0.017), with time spent in food proximity being higher for higher ranking and more central individuals, independently of novelty. Moreover, none of the species differed in the time spent close to the novel versus the familiar side, except for Barbary sheep (Fig. 1), which spent significantly more time close to the novel than to the familiar side (post hoc tests: Barbary sheep, posterior estimate -124.1 [HPDs: -190.8 to -56.8]; dromedary, posterior estimate -18.5 [HPDs: -92.7 to 51.2]; goat, posterior estimate -51.7 [HPDs: -132.2 to 20.5]; red deer, posterior estimate -53.9 [HPDs: -132.6 to 34.7]; Przewalski horse, posterior estimate -53.8 [HPDs: -176.3 to 58.5]; giraffe, posterior estimate -28.7 [HPDs: -123.1 to 72.8]; lama, posterior estimate -87.9 [HPDs: -201.5 to 19.7]; guanaco, posterior estimate -37.7 [HPDs: -145.9 to 72.5]; oryx, posterior estimate -22.1 [HPDs: -143.0 to 100.4]; sheep, posterior estimate -56.8 [HPDs: -142.5 to 37.2]). Finally, session number had no significant effect on the time spent in food proximity (posterior estimate: -4.1 [95% CIs: -24.9 to 17.2], *p* = 0.707).

## Discussion

In this study, we tested neophobic responses to novel objects in 74 subjects of 10 different ungulate species and found differences both within and across species that partially supported our predictions. In particular, more socially integrated (i.e., central) individuals were more neophobic than less central ones, showing a higher latency to approach food closer to novel objects (in line with Prediction 2). However, rank and sex did not predict inter-individual differences in neophobia (in contrast to Predictions 1 and 3). Moreover, species differed in their levels of neophobia, with Barbary sheep being less neophobic than all the other species, and spending a higher proportion of time close to novel objects. Given their socio-ecological characteristics (see below and Table 1), these results support the hypothesis that actual group size is the main driver of group differences in neophobia (in line with Prediction 5), while dietary breadth and domestication played little to no role (in contrast to Predictions 4 and 6).

Our results showed clear inter-individual differences in levels of neophobia. Less central individuals had a lower latency to approach the novel side (as compared to the familiar one), suggesting that individuals being less integrated in their social group are also less neophobic, or perhaps more likely to overcome neophobia to increase their food intake. These results are in line with recent studies on primates showing that less central individuals have a lower probability of retrieving food (Amici et al. 2020; Dell'Anna et al. 2020) and are also more likely to overcome neophobia when access to food is uneven across group members (Amici et al. 2020). Across species, less central individuals may more often have to rely on novel food sources to get a share of resources so that lower neophobia might be selected for. Alternatively, it is possible that different personalities may have complementary functions at the group level, with more neophobic individuals contributing to the maintenance of group cohesion (thus also being more central) and less neophobic individuals contributing to the exploration of novel resources and the spread of the group (see Michelena et al. 2009). In both cases, social integration in the group appears to have a complex encompassing effect on individual fitness, in line with other studies in human (Smith and Christakis 2008; Holt-Lunstad et al. 2010) and nonhuman primates (Silk et al. 2003, 2009, 2010; Schülke et al. 2010; Archie et al. 2014; Dell'Anna et al. 2020).

Our results also showed inter-specific differences in neophobia. In particular, Barbary sheep showed a significant preference for the side with the novel object, as compared to the familiar side. In contrast, all the other species were equally likely to select the novel and the familiar side. Which socio-ecological differences best explain these differences? Barbary sheep are not a domesticated species, they show relatively high levels of dietary breadth in the wild (although lower than other species like goats and red deer), and in the wild they usually live in social groups with an intermediate size (see Table 1). However, the group size of Barbary sheep in the zoo was larger than all the other study species (see Table 1). Therefore, our results provide support for the hypothesis that neophobia might decrease when individuals live in larger groups. These results are in line with findings in other taxa, including birds (Heinrich and Marzluff 1991; Stöwe et al. 2006), primates (Visalberghi and Addessi 2000; Lonsdorf 2006; Tarnaud and Yamagiwa 2008; Gustafsson et al. 2011; Masi et al. 2012), cows (Costa et al. 2014), dogs, and wolves (Moretti et al. 2015). However, more studies are needed to confirm these results. First, it would be especially important to confirm these findings by comparing conspecifics living in similar conditions, but having groups of different size. Second, it would be interesting to compare how individuals living in larger groups (as Barbary sheep in our study) perform when being tested alone. In this way, we could better disentangle whether differences in individual neophobic levels are predicted by the group size in which individuals grow or rather by the group size in which they are tested. Such an approach would be especially interesting considering the ongoing debate over the benefits of individual and group testing of personality in social species (e.g., Magnhagen and Bunnefeld 2009; Webster and Ward 2011).

Overall, our findings confirm sociality as a crucial driver of neophobia in animals. On the one hand, social integration in the group may provide key fitness benefits and thus reduce the potential payoffs that individuals might gain by overcoming neophobia and exploring novelty. On the other hand, larger group sizes may provide more opportunities for social learning, reduce stress levels, and ultimately decrease neophobia. Therefore, sociality appears to provide individuals with significant plasticity in their neophobic responses. Further exploring the link between fitness, sociality, and neophobia in other taxa is surely a rewarding endeavor for future studies. For instance, the inclusion of solitary species or eusocial species might reveal further important effects of other aspects of sociality on individual neophobic responses.

In contrast, we find no support for the hypotheses that neophobia is higher in species that have a wider dietary breadth in the wild (Prediction 4) or that have been domesticated (Prediction 6). At the moment, however, these results should be taken with caution, for several reasons. First, there are yet no standardized methods to collect socio-ecological data across ungulate species: In general, even when the same indexes are used (e.g., Simpson’s index of diversity, number of species fed on), methods to collect data often differ across studies due to objective difficulties when collecting data in the wild. Therefore, direct comparisons across species should always be taken with caution, because different methodological approaches might account for much variation in the results. Second, socio-ecological characteristics may also vary strongly within species, across different groups or populations, so that generalizations should be taken with caution (see e.g., Des Roches et al. 2018). This is no trivial issue, as it is still unclear to what extent socio-ecological factors affect behavior in evolutionary or developmental terms (see e.g., de Waal and Johanowicz 1993; Boesch 2012; Brown et al. 2013). Third, inter-specific differences linked to domestication might have been masked by the fact that all our study animals lived in captivity and have therefore had extensive contact to humans through development, causing a general decrease in neophobia in the study subjects. Several studies have shown that captive individuals are often less neophobic, more explorative and/or innovative than wild conspecifics, likely because they are more often exposed to novel objects and/or have more time and energy to devote to these activities (Benson-Amram et al. 2013; Forss et al. 2015; Lazzaroni et al. 2019; but see e.g., Crane and Ferrari 2017 for evidence that neophobia may actually be higher in captive than wild conspecifics). Fourth, factors other than dietary breadth, group size, or domestication might (also) account for inter-specific differences in neophobia. Predation pressure or environmental variability, for instance, might also predict differences in neophobia. By testing captive individuals, we could control for predation risk in this study, but future studies in the wild should ideally test how differences in predation pressure across and within species might affect individual neophobic response. Finally, it should be noted that different measures of neophobia might provide very different results. For this reason, our study relied on different measures (i.e., latency to approach and eat food, time spent in proximity), and indeed, these provided complementary but not identical results. For instance, the presence of more group members in our study appeared to decrease neophobia when measured as time spent in object proximity, but not when measured as latency to approach or eat food, in line with a previous study on ravens (*Corvus corax*; Brown et al. 2013). In the future, studies using a larger variety of novel stimuli (including acoustic or olfactory ones) and directly manipulating food novelty (e.g., changing food taste and texture) will be especially important.

Overall, our study showed a link between low neophobia and low centrality and also larger group size. More studies on more individuals and species are surely needed to confirm these preliminary results. First, future studies should better control for a variety of potentially confounding factors (e.g., previous exposure to human-made objects, enclosure size, group structure, previous life history of the study animals). Second, our study revealed no significant effect of sex on individual levels of neophobia. In the future, it would be interesting to explore whether the inclusion of more ungulate species would lead to different results, as sex might predict differences in neophobia only in species with larger sexual dimorphism (see e.g., Amici et al. 2019), showing that individuals of the larger sex are more likely to innovate than those of the smaller sex). Third, our study only included captive individuals that had spent their whole life in captivity. Captive conditions, however, might increase individual exposure to novel stimuli during lifetime, perhaps decreasing individual neophobic responses and degrading potential inter-individual and inter-specific differences in neophobia. Therefore, future comparative studies should ideally also include individuals from wild groups, whose socio-ecological characteristics should be directly measured with standardized protocols. Fourth, our study measured neophobic response in two different sessions and found no effect of session number on individuals’ neophobic response. While this suggests that our study subjects consistently responded to the stimuli in this study, future research would especially benefit from including more trials and more stimuli to better measure repeatability of the neophobia responses across trials and contexts, for longer time frames. In line with this, this study explored individual reaction to novel objects, which has been correlated to food neophobia and risk taking in other studies (Coleman and Wilson 1998; Bókony et al. 2012; Greggor et al. 2015). However, neophobia might also strongly vary across contexts (e.g., in foraging versus antipredator contexts, toward physical versus social stimuli; e.g., Coleman and Wilson 1998; Boogert et al. 2006; see Greggor et al. 2015). Therefore, future studies should also better disentangle how these different forms of neophobia are linked to each other and distributed within and across species. These studies will not only be important to understand how neophobia responses are distributed between and within species, but will also have an essential role in conservation and animal welfare, to better predict resilience to human changes, success during reintroduction programs, and/or the effect of enrichment activities in captivity (e.g., Lee n.d.; Dukas and Bernays 2000; Nicolakakis et al. 2003; Reader and Laland 2003; Sol et al. 2005a,b; Ramsey et al. 2007; Lefebvre 2011; Griffin 2016).

## Supplementary Information

Below is the link to the electronic supplementary material.

Supplementary file1 (XLSX 40 KB)

Supplementary file2 (DOCX 20 KB)

## Data Availability

Data and script are made available as supplementary information.

## Code availability

The models are described in details in the text, including the packages used, and the script is provided as supplementary information.
