# Supplementary material for: Neophobia in 10 ungulate species—a comparative approach
Source: Behav Ecol Sociobiol. 2021 Jun 23;75(7):102. doi: 10.1007/s00265-021-03041-0 (PMC8219784; doi:10.1007/s00265-021-03041-0)
Supplement: Supplementary file 2 — Supplementary file2 (DOCX 20 KB) [file 265_2021_3041_MOESM2_ESM.docx]

**Supplementary Data**

**TITLE**: Neophobia in 10 ungulate species – a comparative approach

**SHORT TITLE**: Neophobia in ungulates

**JOURNAL NAME:** Behavioral Ecology and Sociobiology

**AUTHOR NAMES**: Alina Schaffer ^1,6^, Alvaro L. Caicoya ^2,3^, Montserrat Colell ^2,3^, Ruben Holland ^4^, Lorenzo von Fersen ^5^, Anja Widdig ^1,6^, Federica Amici ^1,6*^.

**AFFILIATIONS**:

^1^ Behavioral Ecology Research Group, Institute of Biology, University of Leipzig, Leipzig, Germany

^2^ Department of Clinical Psychology and Psychobiology, Faculty of Psychology, University of Barcelona, Barcelona, Spain

^3^ Institute of Neurosciences, University of Barcelona, Barcelona, Spain

^4^ Zoo Leipzig, Leipzig, Germany

^5^ Nuremberg Zoo, Nuremberg, Germany

^6^ Research Group "Primate Behavioural Ecology", Department of Human Behavior, Ecology and Culture, Max Planck Institute for Evolutionary Anthropology, Leipzig, Germany

* **CORRESPONDING AUTHOR**: Federica Amici, amici@eva.mpg.de

Table S-1. Information about the study subjects (those marked with an asterisk were removed from the analyses, see text).

| **Species** | **Subject** | **Sex** | **Rank** | **Centrality** | **Age** |
| --- | --- | --- | --- | --- | --- |
| Barbary sheep | k01m | male | 0.279 | 0.839 | 3 |
|  | k02f | female | 0.414 | 0.818 | 1 |
|  | k02m | male | 0.149 | 0.713 | 2 |
|  | k03f | female | 0.486 | 0.938 | 8 |
|  | k03m | male | 1.000 | 1.000 | 10 |
|  | k04m | male | 0.481 | 0.833 | 4 |
|  | k05f | female | 0.251 | 0.722 | 16 |
|  | k05m | male | 0.151 | 0.785 | 1 |
|  | k06m | male | 0.740 | 0.906 | 4 |
|  | k07m | male | 0.183 | 0.924 | 1 |
|  | k08m | male | 0.000 | 0.743 | 3 |
|  | k100f | female | 0.255 | 0.769 | 2 |
|  | k100m | male | 0.185 | 0.686 | 2 |
|  | k101f | female | 0.165 | 0.807 | 7 |
|  | k10f | female | 0.142 | 0.994 | 12 |
| Dromedary | beige | female | 0.206 | 1.000 | 1 |
|  | blanc | female | 0.000 | 0.907 | 2 |
|  | claire | female | 0.487 | 0.571 | 12 |
|  | sale | male | 0.142 | 0.925 | 1 |
|  | tache | female | 0.556 | 0.238 | 18 |
|  | touffue | female | 0.232 | 0.723 | 11 |
|  | vieux | male | 1.000 | 0.111 | 27 |
| Giraffe | andrea | female | 0.800 | 0.991 | 12 |
|  | ashanti | female | 0.400 | 1.000 | 17 |
|  | gusti | female | 0.000 | 0.881 | 16 |
|  | jamal | male | 0.400 | 0.878 | 4 |
|  | max | male | 1.000 | 0.842 | 22 |
|  | mosegi | male | 0.400 | 0.922 | 7 |
| Goat (group 1) | m1 | male | 1.000 | 0.967 | 5 |
|  | m74 | female | 0.550 | 0.956 | 5 |
|  | m77 | female | 0.587 | 1.000 | 5 |
|  | m78 | female | 0.216 | 0.848 | 5 |
|  | m79 | female | 0.398 | 0.847 | 5 |
|  | w18 | female | 0.000 | 0.827 | 3 |
|  | w36* |  |  |  |  |
|  | w37* |  |  |  |  |
|  | w38 | female | 0.022 | 0.821 | 3 |
| Goat (group 2) | g1 | female | 0.198 | 1.000 | 1 |
|  | g27 | female | 0.446 | 0.958 | 1 |
|  | g34 | female | 0.336 | 0.880 | 1 |
|  | g34+ | female | 0.892 | 0.750 | 2 |
|  | g35 | female | 1.000 | 0.842 | 2 |
|  | g40 | female | 0.409 | 0.858 | 0 |
|  | g83 | female | 0.000 | 0.817 | 1 |
| Guanaco | hin | male | 0.382 | 0.770 | 6 |
|  | lissita | female | 0.000 | 1.000 | 2 |
|  | lolitha | female | 0.204 | 0.980 | 2 |
|  | phibie | female | 1.000 | 0.778 | 8 |
| Lama | flax | male | 1.000 | 1.000 | 2 |
|  | horst | male | 0.583 | 0.775 | 16 |
|  | krumel | male | 0.031 | 0.895 | 2 |
|  | sancho | male | 0.000 | 0.819 | 6 |
| Oryx | christin | female | 0.559 | 0.916 | 2 |
|  | luisa | female | 0 | 0.784 | 2 |
|  | mortadelo | male | 0.113 | 0.904 | 21 |
|  | ofelia | female | 0.177 | 0.893 | 19 |
|  | rufaro | male | 1.000 | 1.000 | 4 |
| Przewalski horse | lasscio | male | 0.942 | 0.996 | 3 |
|  | raissa | female | 0.000 | 0.854 | 23 |
|  | raja | female | 1.000 | 0.946 | 17 |
|  | ratina | female | 0.573 | 1.000 | 15 |
| Red deer | cornu | male | 0.197 | 0.905 | 1 |
|  | grosnez | female | 0.500 | 0.662 | 9 |
|  | male | male | 1.000 | 0.759 | 9 |
|  | od | male | 0.000 | 1.000 | 1 |
|  | og | male | 0.310 | 0.970 | 1 |
|  | saumon | female | 0.772 | 0.777 | 9 |
|  | verte | female | 0.000 | 0.730 | 9 |
| Sheep | m01* |  |  |  |  |
|  | m42 | male | 1.000 | 0.657 | 1 |
|  | w14 | female | 0.579 | 0.984 | 8 |
|  | w19 | female | 0.493 | 0.960 | 8 |
|  | w20 | female | 0.215 | 0.996 | 4 |
|  | w42* |  |  |  |  |
|  | w49 | female | 0.000 | 0.909 | 1 |
|  | w50 | female | 0.198 | 1.000 | 1 |
|  | w91 | female | 0.395 | 0.838 | 5 |
|  | wgreen | female | 0.054 | 0.892 | 4 |

**Script used for the first model**

library(ape)

library(MCMCglmm)

library(caper)

library(geiger)

library(phytools)

# I read the data (there are no missing values)

setwd("C:/Users/F.Amici/Desktop")

xdata=read.table(file="neo.data.animal.txt",header=T,sep="\t")

# If I want to see my phylogenetic tree (see Upham et al, 2019), subsampled and pruned from vertlife.org:

phylo <- read.nexus("output10000trees.nex")

plot(phylo, layout = 1,

type = "phylogram", use.edge.length = TRUE, node.pos = NULL, show.tip.label = TRUE,

show.node.label = FALSE, edge.color = "black", edge.width = 1, edge.lty = 1, font = 3,

cex = par("cex"), adj = NULL, srt = 0, no.margin = FALSE, root.edge = FALSE,

label.offset = 0, underscore = FALSE,x.lim = NULL, y.lim = NULL, direction = "rightwards",

lab4ut = NULL, tip.color = "black", plot = TRUE, rotate.tree = 0,

open.angle = 0, node.depth = 1, align.tip.label = FALSE)

# To add phylogenetic controls, I read the consensus tree (obtained from TreeAnnotator), and make it ultrametric. I then calculate the ginverse matrix, and include random=~animal in the model

mtree <- read.nexus("consensustree")

mtree.ul <- force.ultrametric(mtree, method=c("nnls", "extend"))

Ainv<-inverseA(mtree.ul)$Ainv

# I construct Model 1, setting 2 priors: one for subject (as I have multiple entries per subject), and one for ~animal (as I include phylogenetic controls); species is included as predictor

prior <- list(R = list(V = 1, nu = 0.002),

G = list(G1 = list(V = 1, nu = 1000, alpha.mu = 0, alpha.V = 1), G2 = list(V=1, nu=0.002, alpha.mu=0, alpha.V=1000)))

model1 <- MCMCglmm(latency.app ~ novelty*species + novelty*rank + novelty*centrality +

novelty*sex + session + trialdur, random=~animal + subject, family="gaussian",

ginverse=list(animal=Ainv), data=xdata, prior=prior, verbose=FALSE, nitt=1000000, burnin=100000, thin=300)

summary (model1)

# Checks (including convergence, autocorrelation and estimations of the posterior distribution)

plot(model1$VCV)

plot(model1$Sol)

autocorr.diag(model1$VCV)

autocorr.diag(model1$Sol)

heidel.diag(model1$VCV)

heidel.diag(model1$Sol)

summary(model1$VCV)

summary(model1$Sol)
